# Optimal threshold of a control parameter for tomotherapy respiratory tracking: A phantom study

**DOI:** 10.1002/acm2.13901

**Published:** 2023-01-12

**Authors:** Keisuke Sano, Masayuki Fujiwara, Wataru Okada, Masao Tanooka, Haruyuki Takaki, Mayuri Shibata, Kenji Nakamura, Yusuke Sakai, Hitomi Suzuki, Kanae Takahashi, Masahiro Tanaka, Koichiro Yamakado

**Affiliations:** ^1^ Department of Radiology Hyogo Medical University Nishinomiya Hyogo Japan; ^2^ Department of Radiotherapy Takarazuka City Hospital Takarazuka Hyogo Japan; ^3^ Department of Biostatistics Hyogo Medical University Nishinomiya Hyogo Japan

**Keywords:** dosimetric accuracy, respiratory tracking, tomotherapy

## Abstract

**Background:**

Radixact Synchrony^®^, a real‐time motion tracking and compensating modality, is used for helical tomotherapy. Control parameters are used for the accurate application of irradiation. Radixact Synchrony^®^ uses the potential difference, which is an index of the accuracy of the prediction model of target motion and is represented by a statistical prediction of the 3D distance error. Although there are several reports on Radixact Synchrony^®^, few have reported the appropriate settings of the potential difference threshold.

**Purpose:**

This study aims to determine the optimal threshold of the potential difference of Radixact Synchrony^®^ during respiratory tumor‐motion‐tracking irradiation.

**Methods:**

The relationship among the dosimetric accuracy, motion tracking accuracy, and control parameter was evaluated using a moving platform, a phantom with a basic respiratory model (the fourth power of a sinusoidal wave), and several irregular respiratory model waveforms. The dosimetric accuracy was evaluated by gamma analysis (3%, 1 mm, 10% dose threshold). The tracking accuracy was measured by the distance error of the difference between the tracked and driven positions of the phantom. The largest potential difference for 95% of treatment time was evaluated, and its correlation with the gamma‐pass ratio and distance error was investigated. The optimal threshold of the potential difference was determined by receiver operating characteristic (ROC) analysis.

**Results:**

A linear correlation was identified between the potential difference and the gamma‐pass ratio (R = –0.704). A linear correlation was also identified between the potential difference and distance error (R = 0.827). However, as the potential difference increased, it tended to underestimate the distance error. The ROC analysis revealed that the appropriate cutoff value of the potential difference was 3.05 mm.

**Conclusion:**

The irradiation accuracy with motion tracking by Radixact Synchrony^®^ could be predicted from the potential difference, and the threshold of the potential difference should be set to ∼3 mm.

## 1 INTRODUCTION

Helical tomotherapy is an image‐guided intensity‐modulated radiotherapy (IMRT) modality for the precise treatment of several types of tumors.^1^ IMRT may result in target under‐dosing owing to the complex interplay between the internal motion of the target and the dynamic dose delivery. In addition, because helical tomotherapy emanates helical irradiation, its accuracy has been reported to be influenced by organ motion during treatment.^2^, ^3^ Therefore, technicians have been recommended to manage the respiratory motion during radiotherapy using motion‐encompassing methods, respiratory‐gated techniques, etc.^4^

Radixact Synchrony^®^ (Accuray, Sunnyvale, CA, USA)—a real‐time motion tracking and compensating modality for helical tomotherapy^5^—is controlled by a model to predict the target location based on a correlation between the internal target position acquired by two‐dimensional (2D) kV x‐ray radiographs and the motion of light‐emitting diode (LED) markers placed on the patient's skin. During the real‐time tracking of tumor motion, several control parameters that manage the prediction model accuracy are used for the stable and accurate application of irradiation. The potential difference (***Potential diff***) threshold is used as one of the control parameters to determine the quality of the prediction model. ***Potential diff*** is an estimation of the maximum standard deviation of the target position that is calculated based on all radiographs in the prediction model and the recent LED markers amplitude data. It is a statistical prediction of the three‐dimensional (3D) distance error and is calculated and logged for each kV‐radiograph acquisition. A more stringent threshold of ***Potential diff*** can ensure the accuracy of the prediction model. However, excessive constraints may cause interruptions during treatment and extension of treatment time. Therefore, an appropriate threshold of ***Potential diff*** must be imposed. Although the extant literature contains several reports about the clinical use and physical verification of irradiation accuracy with Radixact Synchrony^®^, ^6^, ^7^, ^8^ few have reported the appropriate settings of ***Potential diff***.

We conducted a phantom study to evaluate the relationship between ***Potential diff*** and irradiation accuracy for irregular waveforms, and thus determined the appropriate threshold.

## 2 MATERIALS AND METHODS

### 2.1 Treatment, planning, and phantom settings

We operated a tomotherapy device (Radixact X9 version 2.0.1, Accuray, Sunnyvale, CA, USA) and used Accuray Precision v3.1 for IMRT planning. A 6‐MV flattening filter free (FFF) photon beam was used to irradiate the phantom material at a dose rate of 1000 monitor units (MU)/min. A 25‐mm jaw setting was used for this plan. The gantry period was 14.2 s and the treatment time was 80.5 s.

A 2D diode array for IMRT quality assurance (SRS‐MapCHECK, Sun Nuclear Corp, Melbourne, FL, USA) and a radiation therapy test phantom (StereoPHAN, Sun Nuclear Corp, Melbourne, FL, USA) were placed on a one‐dimensional (1D) motion platform (CIRS, Model 008PL, Norfolk, VA, USA). The SRS‐MapCHECK is composed of 1013 n‐type diodes arranged in a 77 × 77 mm^2^ face‐centered array, with each diode spaced 2.47 mm from its four neighbors. The SRS‐MapCHECK was inserted into the StereoPHAN and positioned in the horizontal direction. The platform was rotated by 30° about the Z‐axis to enable motion in the 2D (X‐Y) plane, in accordance with the International Electrotechnical Commission (IEC) 61217.

**FIGURE 1:**
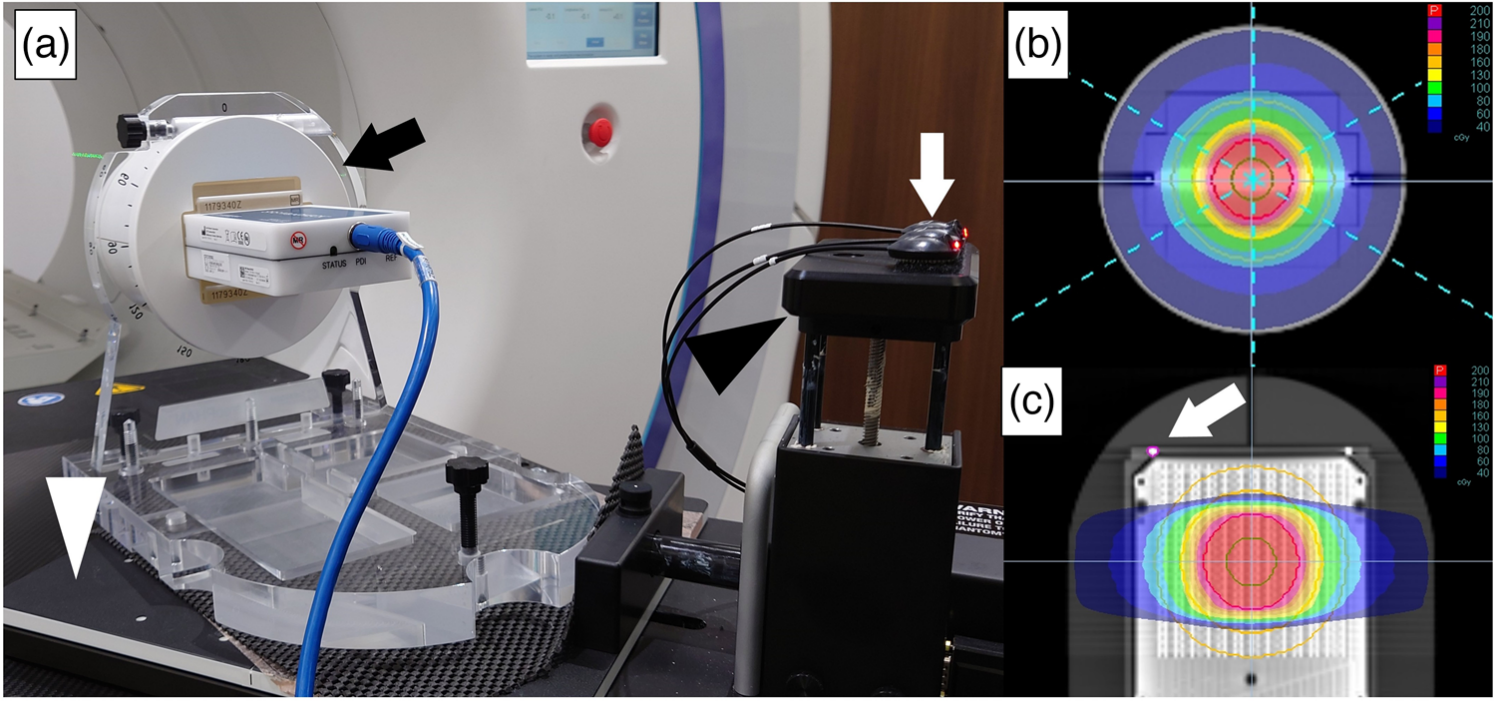
Phantom setup and irradiation plan. (a) Phantom and two‐dimensional (2D) detector were placed on the moving platform: The LED marker was placed on the surrogate stage. The 2D detector, LED marker, surrogate stage, and moving platform were indicated by black arrow, white arrow, black arrowhead, and white arrowhead, respectively. Dose distribution for the (b) transverse section on the isocenter axis and (c) coronal section on the isocenter axis. The marker used for tracking is shown in the pink ROI (indicated by an arrow).

The LED markers, used to produce the surrogate signal, were positioned on the vertical‐direction motion table instead of the patient surface. The phantom setup is illustrated in Figure 1a.

The computed tomography (CT) images of the phantom material were captured by a 16‐slice CT scanner (Aquilion LB, Canon Medical Systems, Otawara, Japan) using the following settings: voltage rating = 120 kV, field‐of‐view (FOV) = 700 mm, and slice thickness = 1 mm. These CT datasets were transferred to the treatment planning software. At the center of the phantom, we generated a spherical volume of interest (VOI) with a diameter of 3 cm and defined it as the planning target volume (PTV). Dose calculations were performed with the entire phantom set to a physical density override of 1.2 g/cm^3^ to compensate for the dose attenuation property. The plan was optimized using IMRT techniques to deliver the prescribed dose to 95 % of the PTV. A dose of 2 Gy/fractions was prescribed (Figure 1b,c).

We used the fiducial free tracking method for the respiratory motion mode and set the tracking target to a metal marker affixed on SRS‐MapCHECK (Figure 1c). The imaging parameters of the kV‐radiograph were 120 kV and 0.8 mAs. Six kV‐radiographs per gantry rotation were acquired at every 60° rotation.

### 2.2 Respiration motion

**FIGURE 2:**
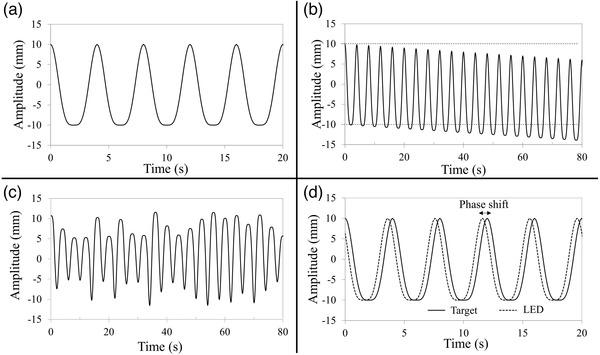
Example of a respiratory waveform input to the moving platform. (a) Basic waveform: modeled as the fourth power of a sinusoidal wave. (b) Baseline shift waveform: baseline shifting at a constant speed (at a shift speed of 3 mm/s). (c) Irregular‐amplitude waveform: amplitude of the basic waveform randomly varies every cycle (with a maximum amplitude variation of 40%). (d) Phase shift waveform: same waveform as the basic waveform, albeit with a shift in the respiration phase between the LED marker and the target (with a 10% phase shift).

In this study, four types of waveforms were created: basic, baseline shift, irregular amplitude, and phase shift. The test phantom, which was placed on the 1D motion platform, reciprocated the motion according to these waveforms. An example of each waveform is depicted in Figure 2a,d. Each respiratory cycle lasted 4 s. The amplitude corresponding to each cycle was ±10 mm, except for the irregular amplitude waveform. An LED marker was placed on the vertical‐direction motion table and translated synchronously with the 1D motion platform. For the baseline shift and irregular amplitude waveforms, the measurements were performed with two types of LED marker movements—phantom‐synchronized motion and phantom‐asynchronized motion. For asynchronized motion, only the phantom was moved with an irregular waveform (baseline shift or irregular amplitude waveform), while the LED markers used the base waveform (fourth power of a sinusoidal wave).

(i) Basic waveform of respiratory motion

The platform motion with the basic waveform was modeled as the fourth power of a sinusoidal wave (Figure 2a). The basic wave function was calculated using the following formula:

(1) $$f\left(t\right)=A\;\mathrm{si}n^{4}\left(2\pi \frac{t}{T}\right),$$

where A and T represent the amplitude of the motion and respiratory cycle, respectively.

(ii) Baseline shift respiratory motion

For the baseline shift respiratory motion, the platform was driven by the basic waveform with baseline shift effects (Figure 2b). The wave function of a baseline shift motion was calculated using the following formula:

(2) $$f\left(t\right)=A\;\mathrm{si}n^{4}\left(2\pi \frac{t}{T}\right)-Bt,$$

where B represents the shift speed, and the measurements were performed by varying the baseline shift speed by 1, 2, and 3 mm/min.

(iii) Irregular amplitude respiratory motion

For the irregular amplitude respiratory motion, the platform was driven using a different basic waveform amplitude every cycle. This amplitude was randomly varied within 10 mm (Figure 2c). The wave function of this type of motion was calculated using the following formula:

(3) $$Ai=10+\left(\mathrm{dmax}\;\times ki\right),$$

where d_max_ and ki represent the maximum variation in the amplitude and a random factor, respectively. The random factor ranged from −1 to +1, and the Mersenne Twister algorithm was used to generate the random numbers. The measurements were performed by varying d_max_ by 10%, 20%, 30%, and 40% of 10 mm.

(iv) Phase shift respiratory motion

For the phase shift respiratory motion, the phantom and the LED marker were translated using the basic waveform, and a respiratory phase shift was generated (Figure 2d). The wave function was calculated using the following formula:

(4) $$f\left(t\right)=A\;\mathrm{si}n^{4}\left(2\pi \frac{t}{T}+\varphi \right),$$

where φ represents the phase shift between the phantom and the LED marker. The phase shift was defined as “positive” when the LED marker moves ahead in time of the platform and “negative” when it moves behind the platform. The measurements were performed by varying the phase shift every 5% from −20% to 20% of the respiratory cycle.

### 2.3 Evaluation of irradiation accuracy

The 2D dose distributions were measured five times for each respiratory motion pattern because the phase shift between the treatment and the phantom motion was different and random for each measurement. Gamma analyses of all measurements were performed using detector control software (SNC Patient Software version 8.2, Sun Nuclear Corp, Melbourne, FL, USA).

The appropriate criteria and tolerances for accuracy control in the case of motion‐tracking irradiation are not specified in guidelines such as AAPM TG‐218.^9^ However, some studies have recommended tighter tolerances than the standard IMRT for SBRT, whereas others had reported the need to impose stringent criteria.^9^, ^10^, ^11^, ^12^ Therefore, we used the following criteria for the gamma analysis: dose difference (DD) = 3%, distance‐to‐agreement (DTA) = 1 mm, absolute dose, and dose threshold = 10%. A measured gamma‐pass ratio of above 90% was considered a pass.

The log files generated after motion‐tracking irradiation were analyzed to verify the accuracy of the prediction model. The predicted positions of the tracking target were read out from the log data in the system and compared with the waveform data used for the phantom motion. The accuracy of motion tracking was measured by the 3D distance error between the tracked position and the driven position of the phantom. These phases were aligned to minimize the root mean square error before analysis. The 3D distance error is expressed as follows:

(5) $$\begin{array}{ccc} & & 3D\;\mathrm{distance}\;\mathrm{error}\;\left(\mathrm{mm}\right)=\\ & & \;\sqrt{{\left(x_{i}-\hat{x_{i}}\right)}^{2}+{\left(y_{i}-\hat{y_{i}}\right)}^{2}+{\left(z_{i}-\hat{z_{i}}\right)}^{2}}, \end{array}$$

where $x_{i}$, $y_{i}$, and $z_{i}$ represent the predicted positions acquired from the log data, and $\hat{x_{i}},\hat{y_{i}},\;$and $\hat{z_{i}}$ the target positions acquired by the platform‐driven waveform. In this study, the maximum value excluding the largest 5% of the 3D distance error during the beam‐on time (*δ_95_*) was evaluated as the accuracy of the prediction model throughout the irradiation. The relationship between dosimetric and prediction model accuracies was evaluated for all types of respiratory motion.

### 2.4 Evaluation of control parameters

***Potential diff*** threshold is one of the control parameters that should be set to determine the quality of the prediction model for Radixact Synchrony^®^. ***Potential diff*** is defined as the maximum standard deviation of the target position to be used for the subsequent model and can predict the 3D error.^13^ The prediction model includes up to 20 radiographs in the model, and ***Potential diff*** is calculated based on all images in the model and recent LED amplitude data. To investigate the effect of ***Potential diff*** on the irradiation accuracy, the phantom was irradiated with the threshold of the ***Potential diff*** set to 10 mm, so that the session would not be interrupted. The maximum value excluding the largest 5% of ***Potential diff*** during beam‐on‐time (*PD_95_*) was evaluated, and its correlation with the gamma‐pass ratio and *δ_95_* was investigated.

### 2.5 Statistical analysis

SPSS Statistics v28.0 (IBM Inc., Armonk, USA) and R software v4.1.0 (R Foundation for Statistical Computing, Vienna, Austria) were used for the statistical analysis. The results were expressed as mean ± standard deviations. The statistical significance was analyzed using the Student's t‐test. A *p*‐value of <0.05 was considered statistically significant. All *p*‐values were two‐sided.

The receiver operating characteristic (ROC) curves were plotted, and the area under the curve (AUC) was calculated to evaluate the discriminative power of ***Potential diff*** for the pass/fail judgment using the gamma analysis. As mentioned in *Evaluation of Irradiation Accuracy*, a measurement with a gamma‐pass ratio of 90% or more was considered a pass. The sensitivity was used to measure the percentage of failed true positive measurements. The maximum Youden's Index was referred to as the optimal cutoff value for accurate respiratory tracking control. The concurrence of *δ_95_* and *PD_95_* was assessed using an intraclass correlation coefficient (ICC) with the two‐way random‐effects model. The ICC scores were interpreted as described by Koo et al.,^14^ with a score of 0–0.50 indicating poor, 0.50–0.75 indicating moderate, 0.75–0.90 indicating good, and a > 0.90 indicating excellent.

## 3 RESULTS

### 3.1 Irradiation accuracy

(i) Basic waveform

The average gamma‐pass ratio for the measurement using the basic waveform was 99.0 ± 1.01%, and *δ_95_* was less than 1 mm (Table 1). This confirmed that the motion‐tracking irradiation for stable respiratory waveforms was performed accurately.

(ii) Baseline shift respiratory motion

**TABLE 1:** Dosimetric and tracking log analysis for various respiratory waveforms

	Variation	LED marker synchronization	Gamma‐pass ratio (%) [3%/1 mm]	*δ_95_* (mm)	*PD_95_* (mm)
Basic waveform		NA	99.0 ± 1.01	0.78 ± 0.23	0.62 ± 0.04
Baseline shift waveform	Shift speed				
	1 mm/min	With	97.9 ± 0.64	0.76 ± 0.03	0.56 ± 0.05
	2 mm/min		98.7 ± 0.86	1.08 ± 0.03	0.51 ± 0.05
	3 mm/min		99.9 ± 0.22	1.54 ± 0.02	0.53 ± 0.13
	1 mm/min	Without	98.0 ± 1.31	1.05 ± 0.09	0.81 ± 0.06
	2 mm/min		98.4 ± 1.73	1.44 ± 0.11	1.32 ± 0.08
	3 mm/min		97.0 ± 1.72	1.67 ± 0.08	2.03 ± 0.08
Irregular‐amplitude waveform	Maximum variation				
	10%	With	99.2 ± 0.94	1.67 ± 0.04	0.63 ± 0.05
	20%		99.5 ± 0.65	0.86 ± 0.05	0.55 ± 0.09
	30%		99.5 ± 0.46	1.20 ± 0.09	0.54 ± 0.07
	40%		99.4 ± 0.70	1.04 ± 0.07	0.58 ± 0.07
	10%	Without	98.0 ± 1.30	1.96 ± 0.09	1.08 ± 0.17
	20%		98.0 ± 0.94	2.15 ± 0.43	2.11 ± 0.23
	30%		91.5 ± 3.31**	2.89 ± 0.15	2.81 ± 0.54
	40%		82.2 ± 5.75**	4.13 ± 0.46	3.32 ± 0.29
Phase‐shift waveform	Phase shift				
	–20%	NA	63.4 ± 7.36**	8.58 ± 1.91	4.86 ± 0.56
	–15%		87.5 ± 7.06*	6.64 ± 1.16	3.75 ± 0.88
	–10%		98.2 ± 1.49	2.75 ± 0.43	2.82 ± 0.19
	–5%		99.4 ± 0.50	1.01 ± 0.07	1.80 ± 0.16
	5%		99.6 ± 0.51	1.07 ± 0.52	1.18 ± 0.04
	10%		99.0 ± 0.75	1.93 ± 0.13	2.57 ± 0.26
	15%		95.5 ± 2.65*	3.83 ± 1.23	4.61 ± 0.54
	20%		78.2 ± 5.77**	6.24 ± 0.84	5.10 ± 1.58

LED: light‐emitting diode. PD95: largest value of potential difference for 95 %. NA: not applicable.

LED marker synchronization: With; the same respiratory waveform as the phantom was used for the LED marker motion. Without; the basic waveform was used for LED marker motion without depending on the target motion.

^*^p‐value < 0.05.

^**^p‐value < 0.01, compared with basic waveform.

**FIGURE 3:**
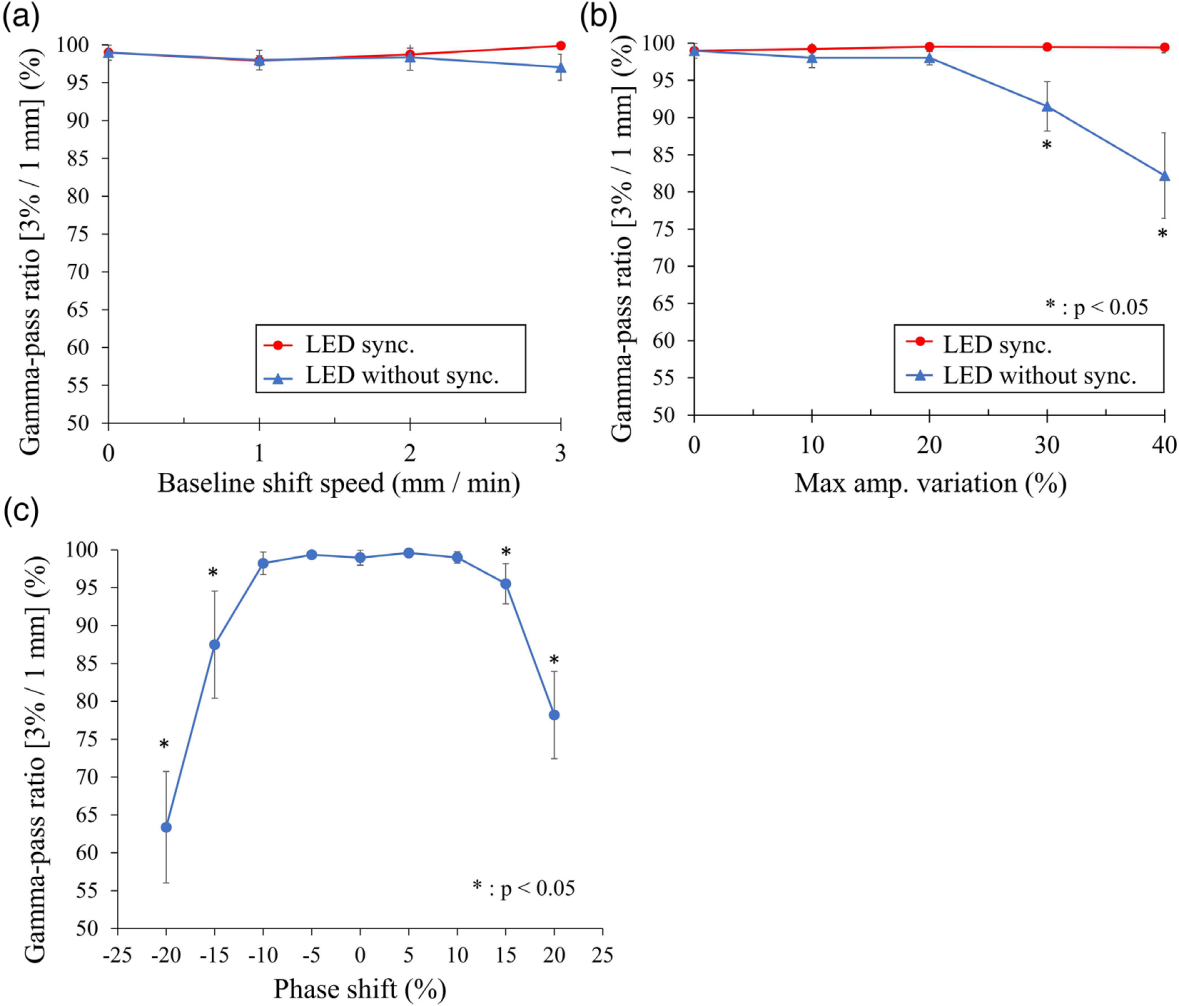
Dosimetric analyses for each respiratory waveform. Dosimetric analysis for (a) baseline shift waveform and (b) irregular amplitude respiratory waveform. LED marker motion with synchronization (LED sync.) and without synchronization (LED without sync.) are represented by red and blue lines, respectively. Statistical analysis is used to compare each condition with a stable waveform. (c) Dosimetric analysis for the phase shift waveform. Phase shift is defined as positive when the LED marker moves ahead of the platform and negative when it moves behind it. Statistical analysis is compared with no phase shift (0%) and each condition. *p*‐value is Student's t‐test. *p < 0.05.

The average gamma‐pass ratio for the measurements using the baseline shift waveform ranged from 97.0 ± 1.72% to 99.9 ± 0.22% and was greater than 90% in all measurements (Table 1). When the baseline shift speed was less than 3 mm/min, no significant decrease in the gamma‐pass ratio occurred, regardless of the synchrony of the phantom and LED marker. However, when the phantom and LED marker were asynchronous, the results for 3 mm/min exhibited a marginally lower pass ratio (Figure 3a).

The *δ_95_* at the 3‐mm/min baseline shift with and without synchronization of the LED marker and target were 1.54 and 1.67 mm, respectively. As with the gamma‐pass ratio, the larger the baseline shift between the target and LED marker motion, the larger was the *δ_95_*.

(iii) Irregular amplitude respiratory motion

The average gamma‐pass ratio for the measurement using the irregular amplitude respiratory waveform was 99.2%–99.5% in the case of the synchronized LED marker. In the case where the LED marker was not synchronized (Table 1 and Figure 3b), the average was 82.2%–98.0%. Even in the case where the amplitude fluctuated, the gamma‐pass ratio did not decrease when the LED marker was synchronized. By contrast, it significantly decreased compared with the basic waveform when the irregular amplitude respiratory motion exceeded 30% with an asynchronous LED marker (91.5% at 30% of the irregular amplitude and 82.2% at 40% of the irregular amplitude, *p* < 0.01 in both cases). The largest value of *δ_95_* was 1.67 mm when the LED markers and phantom motion were synchronized and 4.13 mm when not.

(iv) Phase shift respiratory motion

The average gamma‐pass ratio for the measurement using the phase shift waveform was 63.4%–99.6%. The large phase difference between the LED marker and the target largely affected the irradiation accuracy (Figure 3c). When the phase shift was greater than ± 15%, the gamma‐pass ratio was significantly decreased than that without phase shift. *δ_95_* increased with increasing magnitude of phase shift (6.24 mm at 20% shift and 8.58 mm at −20% shift).

### 3.2 Relationship between the gamma‐pass ratio and *δ_95_*

**FIGURE 4:**
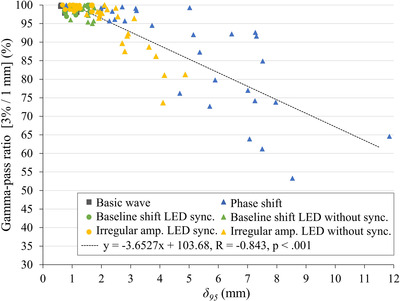
Relationship between gamma‐pass ratio and *δ_95_*. The linear correlation exists between *δ_95_* and the gamma‐pass ratio. In each respiratory waveform, the phantom and LED markers moving with the same waveform input and those moving with different inputs are represented as circles and triangles, respectively.

The relationship between the gamma‐pass ratio and *δ_95_* is illustrated in Figure 4. A linear correlation between the two is observed (R = –0.843, *p* < 0.001).

### 3.3 Control parameters

**FIGURE 5:**
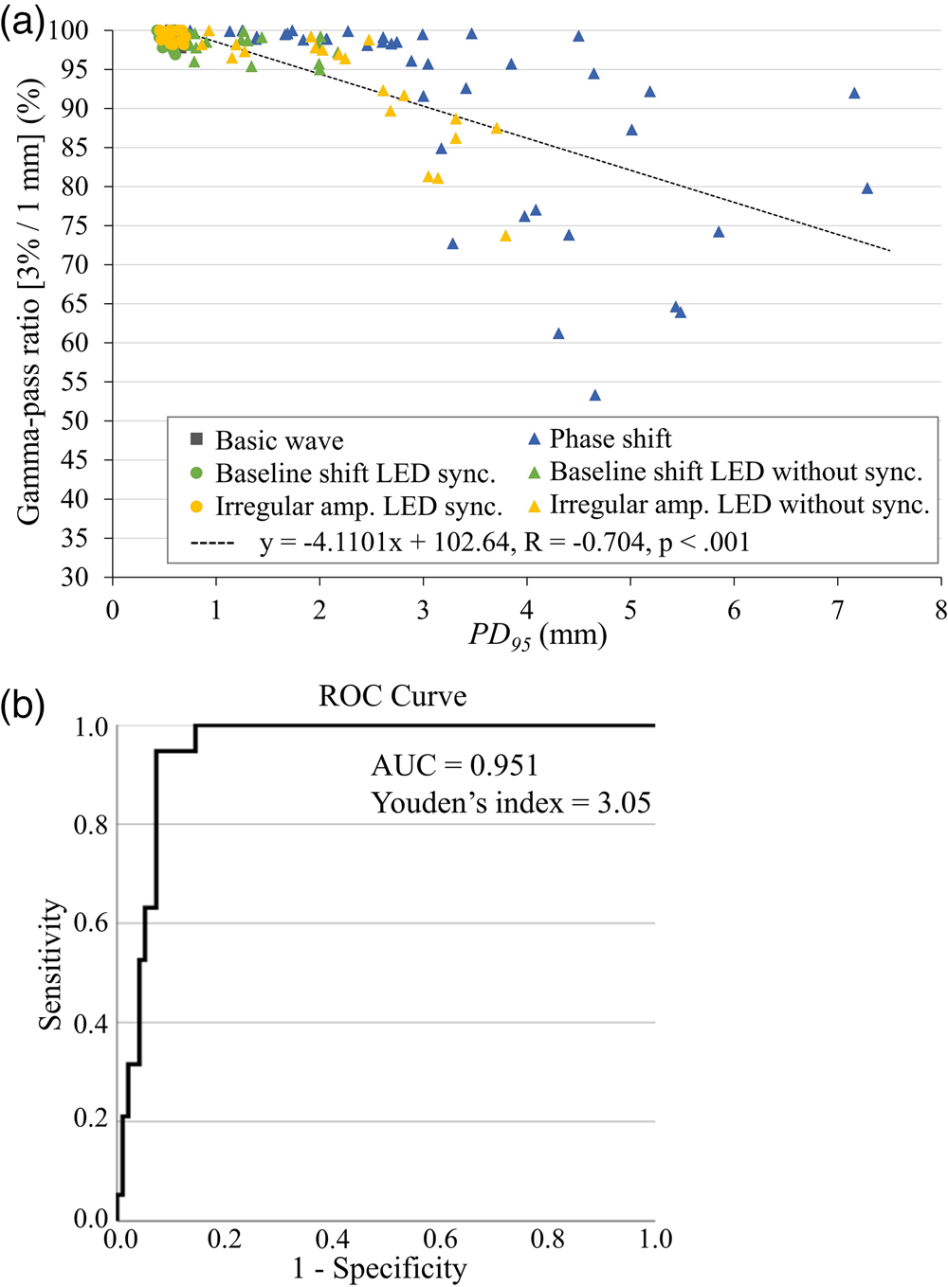
(a) Relationship between maximum ***Potential diff*** for 95% (*PD_95_*) and gamma‐pass ratio. *PD_95_* and gamma‐pass ratio are linearly correlated. In each respiratory waveform, the phantom and LED markers moving with the same waveform input and those moving with different inputs are indicated by circles and triangles, respectively. (b) Receiver operating characteristic (ROC) curve analysis with *PD_95_* as the predictor variable. Sensitivity is used to measure the percentage of true positive failed measurements. The area under the curve for this model is 0.951. The optimal cutoff value of the potential difference is 3.05 mm, according to Youden's index.

*PD_95_* and the gamma‐pass ratio (R = –0.704, *p* < 0.001) were linearly correlated (Figure 5a). However, this correlation was weaker than that between *δ_95_* and gamma‐pass ratio. In particular, the gamma‐pass ratio significantly decreased for a *PD_95_* of more than 3 mm. The ROC curve is depicted in Figure 5b. The AUC for this model was 0.951 (95% CI: 0.914–0.989), indicating that *PD_95_* could discriminate between pass and fail in the gamma analysis. According to Youden's Index, we considered 3.05 mm the optimal cutoff value of ***Potential diff*** for the accurate respiratory tracking control.

**FIGURE 6:**
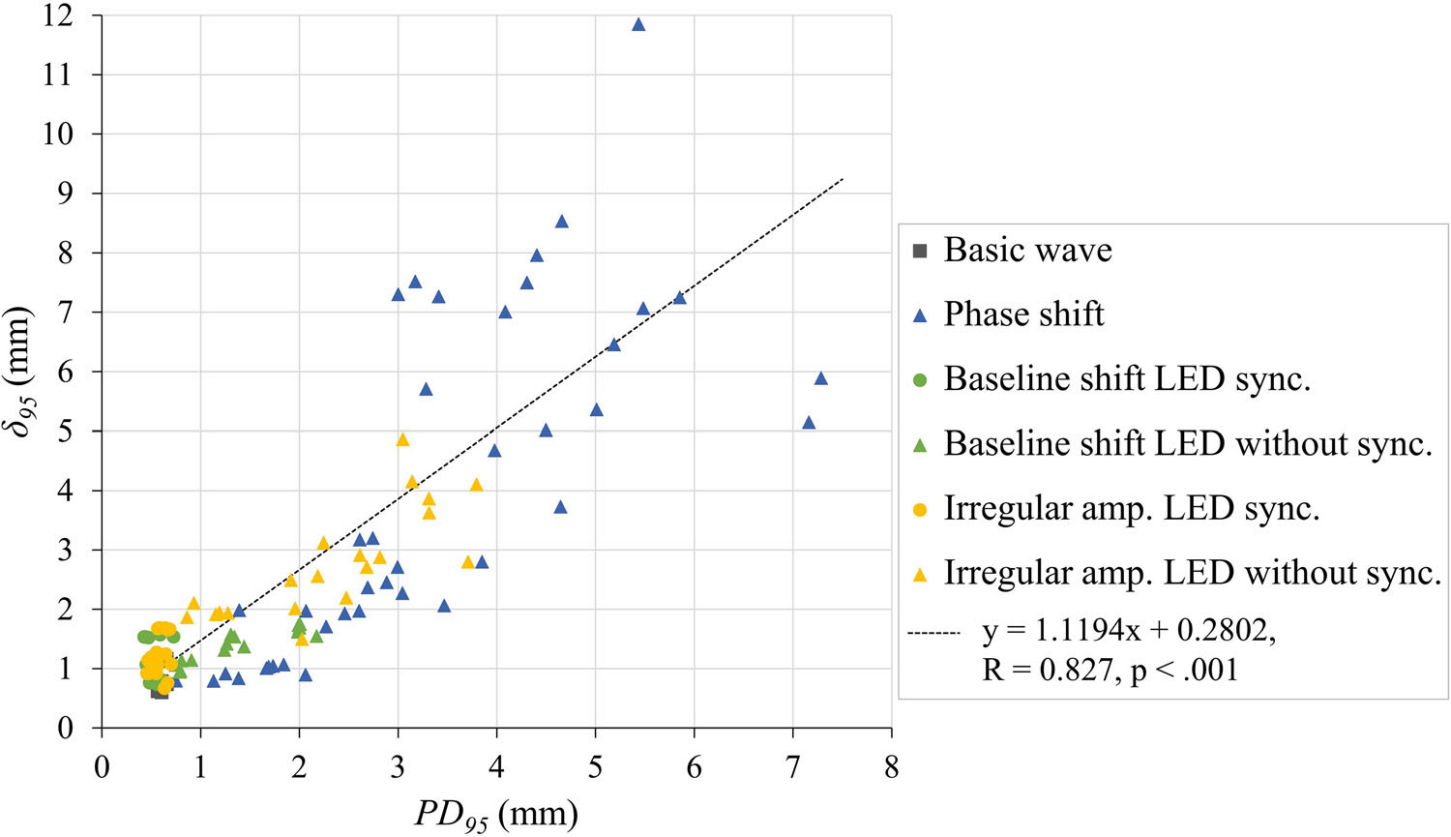
Maximum ***Potential diff*** for 95% (*PD_95_*) versus maximum 3D distance errors for 95% (*δ_95_*). The correlation is approximately linear and shows good agreement (ICC 0.763; 95% CI 0.632–0.845). In each respiratory waveform, the phantom and LED marker moving with the same waveform input are indicated by circles, and those moving with different inputs by triangles.

The relationship between *PD_95_* and *δ_95_* is displayed in Figure 6. The correlation was approximately linear (R = 0.827, *p* < 0.001), indicating strong concurrence (ICC 0.763; 95% CI 0.632–0.845). However, comparing the data of more than 3 mm and those of less than 3 mm for *PD_95_*, the concurrence was significantly lower for the former (ICC 0.222; 95% CI −0.093 to 0.524) than the latter (ICC 0.689; 95% CI 0.499–0.804). In addition, the difference of the ICC between the data with more and less than 3 mm was 0.467 (95% bootstrap CI was 0.262–0.664). Furthermore, as *PD_95_* increased (especially to more than 3 mm), ***Potential diff*** tended to underestimate the prediction model error.

## 4 DISCUSSION

Radixact Synchrony^®^ is a real‐time respiratory tracking and compensation system for the helical tomotherapy modality. ***Potential diff*** threshold is a control parameter that should be used to determine the quality of the prediction model during irradiation. The appropriate setting of this parameter provides the stable and accurate application of irradiation. Therefore, an appropriate threshold must be set for ***Potential diff***. For the baseline shift and irregular amplitude respiratory motion, the results of the gamma analysis with LED marker synchronization did not exhibit a significant decrease. Takao et al. reported that the incidence of baseline shift exceeding 3 mm was 42.1% for the sum of square roots of the three directions within 10 min during radiation treatment.^15^ Dobashi et al. showed that the mean intra‐fractional variation in the peak inhalation position relative to the amplitude in the first respiratory cycle during radiotherapy was 15.5 ± 9.3%.^16^ In this study, no significant effects were observed with baseline shifts of up to 3 mm/min and amplitude changes of 40%. Therefore, the effect of respiratory variability may be minor, and Radixact Synchrony^®^ can accurately track the motion of the tumor.

However, the dosimetric accuracy declined when the correlations between the LED marker and the target changed (Figure 3a,b). Particularly, compensating for abrupt changes, such as an irregular amplitude waveform is more difficult than for slow changes, such as a baseline shift. Radixact Synchrony^®^ constructs and updates a prediction model from the movement of the LED marker and the tumor position obtained from intermittent kV‐radiographs. Therefore, the modality may not recognize the changes in the correlation between the LED marker and the tumor position in the interval between the kV shots. A previous study reported that external chest motions were strongly correlated with internal tissue motions.^17^ However, Malinowski et al. reported that the relationship between the chest and tumor positions in patients with pulmonary and pancreatic tumors changed in 63% of the fractions.^18^ Awareness of movements that may alter the correlation between the tumor and the LED marker, such as deep inhalation, muscle movements, and peristalsis, is necessary.

On the one hand, in the case with a phase shift of less than 10% in the phase shift respiratory motion, the gamma‐pass ratio and *δ_95_* did not change. However, in the case with a shift of more than ± 15%, the gamma‐pass ratio decreased. Ferris et al. discovered that on Radixact Synchrony, with an LED marker phase shift of 20%, the largest value of *δ_95_* was a relatively small 2.9 mm for the liver and 2.3 mm for the lungs.^8^ By contrast, Akino et al. reported a similar experiment with Cyberknife Synchrony^®^, wherein they observed extremely large tracking errors when the LED marker delayed in the respiratory phase and δ_95_ was above 9 mm with a 15% delay phase shift.^19^ In this study, the largest value of *δ_95_* was 8.58 mm at a phase shift of –20%. Moreover, in this study, *δ_95_* was phase‐optimized in the analysis. Therefore, the effect of the target tracking phase shift was eliminated. The impact on the prediction model accuracy was possibly greater than that in the previous report^8^ because the threshold of ***Potential diff*** was set as approximate in this study to examine changes in the parameters.

The correlation between *δ_95_* and gamma‐pass ratio was approximately linear (Figure 4); therefore, the dosimetric accuracy can be estimated from the value of the prediction model error. The accuracy of the prediction model greatly affects the delivery of accurate dose distributions, and it is important to control the prediction model accuracy.

Although studies have used ***Potential diff*** as an index of accuracy of the prediction model for research and during treatment, none have reported its relationship with the irradiation accuracy and the appropriate settings. In this study, most measurements where *PD_95_* was less than 3 mm passed the gamma analysis, whereas 3.05 mm was considered the optimal cutoff value based on the ROC analysis. In addition, ***Potential diff*** concurred with *δ_95_* in the case where *PD_95_* was less than approximately 3 mm. Therefore, ***Potential diff*** threshold is a critical control parameter for estimating the accuracy of treatment. However, as *PD_95_* increased, especially to more than 3 mm, ***Potential diff*** tended to underestimate the prediction model error. ***Potential diff*** is calculated using all radiographs included in the prediction model; however, the radiographs are excluded from the model building when the target was poorly recognized or deviated greatly from the predicted location. Thus, only radiographs shot at specific instants of time and angles that the current prediction model could predictable were used for building the model and calculating ***Potential diff***; this selection of radiographs may have caused an underestimation of the prediction model error. From these results, we recommend that technicians use approximately 3 mm as the threshold of ***Potential diff***.

Radixact has another control parameter called Measured delta, a measure of the accuracy that the prediction model was in predicting the target location in the most recent radiographs. It has been reported that the measured delta is more responsive to errors than ***Potential diff***.^20^ However, there is the disadvantage that the measured delta is not calculated when fiducials or targets are not detected due to low image quality or when the detected target location is far from the expected target location. In this study, measurements that showed a decrease in pass rate resulted in many deficiencies in the measured delta values in the log data; therefore, the measured delta was not used in the evaluation. The appropriate control parameters may depend on the factors or motion causing the error and require further validation.

This study provides a valuable resource for the optimization of the parametric settings by investigating the effects of various respiratory waveforms on the irradiation accuracy and control parameters. In this study, a simple spherical target was used as the PTV, and plan parameters, such as gantry speed, were not evaluated. However, inpatient treatment, many factors such as tumor shape, image recognition, and plan are expected to affect tracking accuracy and control parameters. Therefore, it is necessary to investigate more complex conditions and to optimize the threshold of control parameters to the governing patient and institutional policy, such as the PTV margins. However, a ***Potential diff*** greater than 3 mm causes it to deviate from the irradiation accuracy. Therefore, we do not recommend a wide margin of deviation from the threshold of ***Potential diff***.

## 5 CONCLUSIONS

The Radixact Synchrony^®^ modality accurately tracks the target for various respiratory model waveforms. However, the irradiation accuracy decreased when the correlation between the LED marker and the target motion deviated. ***Potential diff*** threshold is a critical control parameter for predicting the decrease in accuracy and accuracy control during motion‐tracking irradiation. However, as ***potential diff*** increased, the prediction model error was underestimated. We recommend a threshold of approximately 3 mm for ***Potential diff***.

## AUTHOR CONTRIBUTION

Keisuke Sano, Masayuki Fujiwara, Wataru Okada, Masao Tanooka, Haruyuki Takaki, Hitomi Suzuki, Masahiro Tanaka, and Koichiro Yamakado were involved in study design and data interpretation. Keisuke Sano, Wataru Okada, Mayuri Shibata, Kenji Nakamura, and Yusuke Sakai were involved in the data acquisition and analysis. Kanae Takahashi developed the statistical analysis plan and conducted statistical analyses. Keisuke Sano drafted the original manuscript. All authors critically revised the report, commented on drafts of the manuscript, and approved the final report.

## CONFLICT OF INTEREST

None.
